# Etiology, histology, and long-term outcome of bilateral testicular regression: a large Belgian series

**DOI:** 10.1093/hropen/hoad047

**Published:** 2023-12-01

**Authors:** L J W Tack, C Brachet, V Beauloye, C Heinrichs, E Boros, K De Waele, S van der Straaten, S Van Aken, M Craen, A Lemay, A Rochtus, K Casteels, D Beckers, T Mouraux, K Logghe, M Van Loocke, G Massa, K Van de Vijver, H Syryn, J Van De Velde, E De Baere, H Verdin, M Cools

**Affiliations:** Department of Internal Medicine and Pediatrics, Ghent University, Pediatric Endocrinology Service, Ghent University Hospital, Belgium, Ghent; Université libre de Bruxelles (ULB), Hôpital Universitaire de Bruxelles (H.U.B), Hôpital Universitaire des Enfants Reine Fabiola (HUDERF), Paediatric Endocrinology Unit, Brussels, Belgium; Department of Pediatric Endocrinology, Université Catholique de Louvain—UCLouvain, Brussels, Belgium; Université libre de Bruxelles (ULB), Hôpital Universitaire de Bruxelles (H.U.B), Hôpital Universitaire des Enfants Reine Fabiola (HUDERF), Paediatric Endocrinology Unit, Brussels, Belgium; Université libre de Bruxelles (ULB), Hôpital Universitaire de Bruxelles (H.U.B), Hôpital Universitaire des Enfants Reine Fabiola (HUDERF), Paediatric Endocrinology Unit, Brussels, Belgium; Department of Internal Medicine and Pediatrics, Ghent University, Pediatric Endocrinology Service, Ghent University Hospital, Belgium, Ghent; Department of Internal Medicine and Pediatrics, Ghent University, Pediatric Endocrinology Service, Ghent University Hospital, Belgium, Ghent; Department of Internal Medicine and Pediatrics, Ghent University, Pediatric Endocrinology Service, Ghent University Hospital, Belgium, Ghent; Department of Internal Medicine and Pediatrics, Ghent University, Pediatric Endocrinology Service, Ghent University Hospital, Belgium, Ghent; Department of Pediatrics, AZ Turnhout, Turnhout, Belgium; Department of Pediatric Endocrinology, University Hospitals Leuven, Leuven, Belgium; Department of Development and Regeneration, KU Leuven, Leuven, Belgium; Department of Pediatric Endocrinology, University Hospitals Leuven, Leuven, Belgium; Department of Development and Regeneration, KU Leuven, Leuven, Belgium; Department of Pediatric Endocrinology, Université catholique de Louvain, CHU UCL Namur, Yvoir, Belgium; Department of Pediatric Endocrinology, Université catholique de Louvain, CHU UCL Namur, Yvoir, Belgium; Department of Pediatric Endocrinology, AZ Delta, Roeselare, Belgium; Department of Pediatric Endocrinology, AZ Delta, Roeselare, Belgium; Department of Pediatric Endocrinology, Jessa Ziekenhuis, Hasselt, Belgium; Department of Pathology, Ghent University Hospital, Ghent, Belgium; Center for Medical Genetics, Ghent University Hospital, Ghent, Belgium; Department of Biomolecular Medicine, Ghent University, Ghent, Belgium; Department of Internal Medicine and Pediatrics, Ghent University, Pediatric Endocrinology Service, Ghent University Hospital, Belgium, Ghent; Center for Medical Genetics, Ghent University Hospital, Ghent, Belgium; Center for Medical Genetics, Ghent University Hospital, Ghent, Belgium; Department of Biomolecular Medicine, Ghent University, Ghent, Belgium; Center for Medical Genetics, Ghent University Hospital, Ghent, Belgium; Department of Internal Medicine and Pediatrics, Ghent University, Pediatric Endocrinology Service, Ghent University Hospital, Belgium, Ghent

**Keywords:** testicular regression, vanished testes, differences of sex development (DSD), hormone replacement therapy, long-term outcome, *DHX37*

## Abstract

**STUDY QUESTION:**

What is the long-term outcome of individuals born with bilateral testicular regression (BTR) in relation to its underlying etiology?

**SUMMARY ANSWER:**

Statural growth and pubertal development are adequate with incremental doses of testosterone replacement therapy (TRT); however, penile growth is often suboptimal, especially in those with a suspected genetic etiology (i.e. heterozygous *DHX37* variants) or a micropenis at birth.

**WHAT IS KNOWN ALREADY:**

BTR is a rare and poorly understood condition. Although a vascular origin has been postulated, heterozygous missense variants in *DHX37* have been attributed to the phenotype as well. How these various etiologies impact the clinical phenotype, gonadal histology and outcome of BTR remains unclear.

**STUDY DESIGN, SIZE, DURATION:**

For this cross-sectional study, individuals with BTR were recruited in eight Belgian pediatric endocrinology departments, between December 2019 and December 2022. A physical exam was performed cross-sectionally in all 17 end-pubertal participants and a quality of care questionnaire was completed by 11 of them. Exome-based panel testing of 241 genes involved in gonadal development and spermatogenesis was performed along with a retrospective analysis of presentation and management. A centralized histological review of gonadal rests was done for 10 participants.

**PARTICIPANTS/MATERIALS, SETTING, METHODS:**

A total of 35 participants (33 with male, 1 with female, and 1 with non-binary gender identity) were recruited at a mean age of 15.0 ± 5.7 years.

**MAIN RESULTS AND THE ROLE OF CHANCE:**

The median age at presentation was 1.2 years [0–14 years]. Maternal gestational complications were common (38.2%), with a notably high incidence of monozygotic twin pregnancies (8.8%). Heterozygous (likely) pathogenic missense variants in *DHX37* (p.Arg334Trp and p.Arg308Gln) were found in three participants. No other (likely) pathogenic variants were found. All three participants with a *DHX37* variant had a microphallus at birth (leading to female sex assignment in one), while only six of the remaining 31 participants without a *DHX37* variant (19.4%) had a microphallus at birth (information regarding one participant was missing). Testosterone therapy during infancy to increase penile growth was more effective in those without versus those with a *DHX37* variant. The three participants with a *DHX37* variant developed a male, female, and non-binary gender identity, respectively; all other participants identified as males. TRT in incremental doses had been initiated in 25 participants (median age at start was 12.4 years). Final height was within the target height range in all end-pubertal participants; however, 5 out of 11 participants (45.5%), for whom stretched penile length (SPL) was measured, had a micropenis (mean adult SPL: 9.6 ± 2.5). Of the 11 participants who completed the questionnaire, five (45.5%) reported suboptimal understanding of the goals and effects of TRT at the time of puberty induction. Furthermore, only 6 (54.5%) and 5 (45.5%) of these 11 participants indicated that they were well informed about the risks and potential side effects of TRT, respectively. Histological analysis of two participants with *DHX37* variants suggested early disruption of gonadal development due to the presence of Müllerian remnants in both and undifferentiated gonadal tissue in one. In eight other analyzed participants, no gonadal remnants were found, in line with the BTR diagnosis.

**LIMITATIONS, REASONS FOR CAUTION:**

The limitations of this study include the relatively small sample size (n = 35) and the few individuals with *DHX37* variants (n = 3). Furthermore, data on the SPL were often missing, due to this being undocumented or refused by participants.

**WIDER IMPLICATIONS OF THE FINDINGS:**

TRT provides adequate statural growth, even when initiated in late adolescence, thus providing time for physicians to explore the patients’ gender identity if needed. However, sufficient and understandable information regarding the effects and side effects of TRT is required throughout the management of these patients. SPL remains suboptimal in many individuals and could be improved by TRT during infancy to mimic the physiological mini-puberty. An environmental origin in some participants is supported by the high incidence of gestational complications (38.2%) and by the three monozygotic twin pregnancies discordant for the BTR phenotype. Individuals with a heterozygous *DHX37* variant have a more severe phenotype with severely restricted penile growth until adulthood. Histological analysis confirmed *DHX37* as a gonadal development, rather than a BTR-related, gene.

**STUDY FUNDING/COMPETING INTEREST(S):**

Funding was provided by the Belgian Society for Pediatric Endocrinology and Diabetology (BESPEED) and by Ghent University Hospital under the NucleUZ Grant (E.D.B.). M.C. and E.D.B. are supported by an FWO senior clinical investigator grant (1801018N and 1802220N, respectively). The authors report no conflicts of interest.

**TRIAL REGISTRATION NUMBER:**

N/A.

WHAT DOES THIS MEAN FOR PATIENTS? Testicular regression is a rare condition in which one or both testicles regress during pregnancy or shortly after birth. It has a similar presentation as the more common undescended testicles, which can pose difficulties for physicians in diagnosing the condition. As both testicles are absent, testosterone replacement therapy is needed in early adolescence to induce and support puberty, and it needs to be maintained through adulthood. However, guidelines regarding the counseling and treatment of these individuals are lacking. In this work, we provide long-term data regarding the diagnosis, management, and results of genetic work-up for 35 individuals with bilateral testicular regression. The exact timing of puberty induction appears of little importance for pubertal growth, providing time for patients and physicians to explore the patient’s needs and wishes. Our end-pubertal cases clearly indicated a need for comprehensive counseling regarding the goals, options, and potential side effects of testosterone therapy, and most men choose the testes prostheses when this option is proposed by their doctor. Genetic work-up can aid counseling, specifically if it screens for *DHX37* variants which present as a different clinical entity with impaired gonadal development as opposed to testicular regression. Penile growth is precarious in a subgroup of men with bilateral testicular regression, especially in those with a *DHX37* variant.

## Introduction

In testicular regression (TR), sometimes called vanishing testis syndrome or congenital bilateral anorchia, one or both testicles regress during fetal or early neonatal life. Patients typically present at birth with fused labioscrotal folds, in which no testes can be palpated. In some cases, there is a micropenis, but usually no hypospadias (Pirgon and Dündar, 2012). This situation accounts for ∼5% of cryptorchidism cases, reflecting an incidence of 1/1250 to 1/2000 males (Spires *et al.*, 2000). During laparoscopy, a blind-ending spermatic cord is found, with usually a small fibrotic nodulous testicular rest, sometimes called a ‘nubbin’ (Law *et al.*, 2006). In a minority of cases (0–16%), viable germ cells or seminiferous tubules are reported in the testicular remnant (Pirgon and Dündar, 2012). Due to the hypothetical risk for malignant degeneration of these cells, based on reports of cryptorchidism cases, some physicians prefer to remove these remnants, especially when in an abdominal position (Campbell, 1942; Rozanski *et al.*, 1996; Nataraja *et al.*, 2018).

Regression of Müllerian duct derivatives, induced by anti-Müllerian hormone (AMH), commences at the 8th week of fetal development and is completed at 10 weeks. Concurrently, masculinization of the external genitalia, triggered by testosterone, occurs between the 10th and 16th weeks of gestation, with continued penile growth until term (Cools and Köhler, 2019). The characteristic clinical presentation of TR is in line with its name and signifies that the testes have indeed developed before regressing. The exact timing of regression, and whether or not the occurrence is unilateral or bilateral, will determine the degree of virilization of the 46,XY fetus (Liu *et al.*, 2018; Baskin *et al.*, 2018). However, as the early stages of male genital development, including Müllerian duct regression and urethral formation, remain unaffected, patients will present with a normal or small penis, orthotopic urethra, and absent Müllerian remnants. Conversely, testicular dysgenesis is associated with early disruption, resulting in patients presenting with Müllerian remnants and hypospadias. Nevertheless, there are rare cases where patients exhibit overlapping clinical features of both conditions. The current view is that ‘46,XY gonadal dysgenesis and TR can be regarded as a continuum of phenotypes due to errors in testis determination and maintenance’ (McElreavey *et al.*, 2020).

Although a multifactorial origin has been suggested, the exact etiology of TR remains controversial (Cools *et al.*, 2018). The hypothesis of a vascular origin was initially widely accepted and was based on histopathologic findings by Smith *et al.* (1991), revealing relatively normal spermatic cord elements without viable testicular tissue, but with fibrovascular tissue containing dystrophic calcifications and haemosiderin depositions. Since then, several studies have tried to identify the underlying genetic factors by screening for pathogenic variants in known differences of sex development (DSD)-related genes, without success (Vinci *et al.*, 2004; Brauner *et al.*, 2011). There have only been two reports of sporadic cases in which Y-chromosome microdeletions and a heterozygous missense variant in *NR5A1* were proposed to be the cause of bilateral testicular regression (BTR) (Calogero *et al.*, 2001; Philibert *et al.*, 2007). A large study by McElreavey *et al.* (2020) discovered heterozygous missense variants in the *DEAH-Box Helicase 37* (*DHX37*) gene in 4 of 16 children with BTR (including one case of unilateral regression), suggesting that genetics could be more important in the etiology of TR than previously thought. Since then, *DHX37* variants have been reported in BTR and 46,XY gonadal dysgenesis in other studies as well (Buonocore *et al.*, 2019; da Silva *et al.*, 2019). The incidence of *DHX37* pathogenic variants in 46,XY gonadal dysgenesis has been estimated to be 10–15% (Elzaiat *et al.*, 2022). The exact mechanism by which the ribosomal RNA helicase DHX37 causes TR remains elusive; however, a link with inappropriate WNT signaling has been suggested (McElreavey *et al.*, 2020; Zidoune *et al.*, 2021). Since only missense variants have so far been reported in BTR, the known variants may cause the phenotype through a gain-of-function mechanism (McElreavey *et al.*, 2022). Patients with BTR require lifelong endocrine follow-up and sex steroid replacement therapy to induce and support puberty and prevent long-term complications of hypogonadism (e.g. osteoporosis and cardiovascular disease) (Carsote *et al.*, 2016; Fouatih *et al.*, 2019). However, little is known regarding their long-term outcome.

This study aimed to assess the endocrine management and outcome of patients with BTR, diagnosed and treated in Belgian pediatric endocrinology tertiary care centers. In addition, we explored the underlying genetic mechanisms in a cohort of patients with BTR using a whole exome sequencing (WES)-based panel of known DSD, male infertility, and hypogonadism genes and reviewed the available histological tissues of gonadal remnants.

## Materials and methods

### Recruitment

Belgian centers with a pediatric endocrinology department were contacted through the Belgian Society for Pediatric Endocrinology and Diabetology (BESPEED) to enroll patients with BTR in this cross-sectional study. Patients with coronal or more proximal hypospadias were excluded, as these phenotypes point toward a diagnosis of 46,XY gonadal dysgenesis rather than BTR. Minor forms (e.g. glandular hypospadias) were not excluded. Eligible patients were invited to have a single cross-sectional assessment at the pediatric endocrinology department when they were adults, or could participate during their regular follow-up visits when they were children or adolescents. The visits took place between December 2019 and December 2022.

### Ethics

Written consent was obtained from all participants and their parents (if the patient was <18 years old) prior to participation. The study was approved by the local ethics boards (B670201836824).

### Retrospective data and exams

Retrospective data were collected from the patient’s files when available. Retrospective data included details of gestation and birth, medical history, initial diagnosis and management during childhood, details of hormone replacement therapy (HRT), as well as surgical and growth data. Target height range was calculated using the following formula:

$$\mathrm{Target}\;\mathrm{height}\;\mathrm{range}\;\left(\mathrm{cm}\right)=\;\frac{\mathrm{Hf}\;+\;\mathrm{Hm}\;+\;13}{2}\;\pm 8$$

Hf is the height of the father and Hm is the height of the mother.

All participants underwent a physical exam including measurement of height and stretched penile length (SPL). A micropenis was defined as 2.5 cm for neonates and 9 cm for adult men (Van Der Straaten *et al.*, 2020; Tack *et al.*, 2022). The latter exam could be refused by the participant. End-pubertal cases were asked to fill in a quality of care questionnaire that has previously been used in the DSD-LIFE study (Rapp *et al.*, 2021), surveying the information they received regarding their condition and testosterone replacement therapy (TRT). Five-point Likert scales were grouped as: 4 or 5 as good/satisfied, 3 as neutral, and 1 or 2 as insufficient/dissatisfied.

### Genetics

In all participants, blood sampling and subsequent DNA extraction were performed for WES.

Details of the genes (n = 241) included in the WES panel and the filtering process of variants are presented in Supplementary Tables S1 and S2. The American College of Medical Genetics guidelines (Richards *et al.*, 2015) were used to classify variants. Parental blood samples were requested for segregation analysis by Sanger sequencing when a (likely) pathogenic variant was identified. Disease-causing oligogenic variant combinations were sought using the Oligogenic Resource for Variant AnaLysis (ORVAL) online platform (Renaux *et al.*, 2019). Digenic variant combinations predicted to be at least 95%-likely disease-causing were withheld and further filtered through literature searches of gene expression and function in male genital and gonadal tissues.

### Pathology

Gonadal remnants and adnexes obtained at laparoscopic exploration and stored as formalin-fixed paraffin-embedded material were reviewed, based on hematoxylin/eosin staining.

### Statistics

Statistical software package of IBM SPSS^©^ version 27.0 (Armonk, NY., USA) was used. A Shapiro–Wilk test was used to test for normality. Two-sided Pearson correlation coefficients were calculated for the association of peak height velocity (PHV), total pubertal growth, delta target height and adult height with the age at start of TRT.

## Results

### Participants

There were 35 participants recruited at eight centers, at a mean and median age of 15.0 ± 5.7 and 16.3 years (IQR: 5.5 years), respectively. One participant had been assigned as female at birth and had developed a female gender identity. The other 34 participants had been raised male, of whom one later identified as non-binary.

### Pregnancy and birth

Details regarding pregnancy and birth were retrospectively collected and are presented in Table 1. These data were (partially) missing in two cases. Gestational complications had occurred in 13 (38.2%) pregnancies. Of note, three monozygotic twin pregnancies and one triplet pregnancy of unknown zygosity were reported. All of the monozygotic twin pairs had experienced twin-to-twin transfusion. Of note, the twins were discordant for the TR phenotype. Two participants had the highest birth weight and one had the lowest birthweight of the twin pair. Severe obstetric complications had occurred in four pregnancies (11.8%). In total, gestational and perinatal complications were documented in 17 participants (50%).

**Table 1.** Data of conception, pregnancy, and birth of the whole cohort (n = 35).

Conception, pregnancy, and birth	
Birth weight SDS (mean ± SDS)	−0.34 ± 0.89 (n = 33)
Birth length SDS (mean ± SDS)	−0.28 ± 0.81 (n = 30)
Gestational age [median (IQR)]	40.0 weeks (2.0) (n = 33)
Small for gestational age	2/33 (6.1%)
Large for gestational age	0/33 (0%)
Preterm birth	5/33 (15.2%)
Use of ART	2/33 (6.1%)
	IVF: 1/33 (3.0%)
	ICSI: 1/33 (3.0%)
Consanguinity	2/35 (5.7%)
Severe gestational complications	13/34 (38.2%)
Twin pregnancy	5/34 (14.7%)
	Monozygotic twin: n = 3
	Dizygotic twins: n = 1
	Triplets (unknown zygosity): n = 1
Pre-eclampsia	1/34 (2.9%)
Gestational diabetes	1/34 (2.9%)
Maternal substance abuse*	1/34 (2.9%)
Other^+^	6/34 (17.6%)
Obstetric complications	4/34 (11.8%)
Pregnancy or obstetric complication	17/34 (50.0%)

SDS: standard deviation score;  maternal substance abuse*: illegal drug and/or alcohol abuse; other^+^: includes severe maternal anemia with need for transfusions, cytomegalovirus infection (n = 2), severe hydronephrosis with IUGR, amniocentesis and placental abruption; obstetric complications: admission at neonatal intensive care (unrelated to diagnostic work-up or suspicion of congenital adrenal hyperplasia) or emergency C-section.

### Presentation and prepubertal management

Participants presented at their respective pediatric endocrinology departments at a median age of 1.2 years (range: 0–14 years) (Table 2); only 6 of the 35 (17.1%) presented in the neonatal period. In none of the participants, gonads could be palpated in the labioscrotal folds or in the inguinal region, at initial presentation nor at any later time point. Small testicular remnants (‘nubbins’) were palpated in inguinal or labioscrotal position in five participants. Measurement of SPL at initial presentation was available for 12 participants. Nine participants (26.5%) were diagnosed by a pediatric endocrinologist as having a micropenis (Table 2). Participant 2 with the most severe phenotype had presented at birth with a small phallic structure (dimensions undocumented), epispadic urethra, posterior labioscrotal fusion, and bilateral unpalpable testes. This participant had been registered female at birth. Unfortunately, a precise description of the external genitalia at birth or an external genitalia score (EGS) (Van Der Straaten *et al.*, 2020) was often lacking in historical records. The diagnosis of BTR was confirmed using AMH dosage, hCG stimulation test, and/or surgery, depending on the preference at the center and the age at diagnosis, with a slight preference for surgery (22.9%, 28.6%, and 34.5%, respectively). However, a combination of AMH measurement, hCG stimulation test, and/or surgery was used in 5/35 participants (14.3%) and 15 (50.0%) underwent additional confirmatory tests after the diagnosis was made (Table 2). Nine participants received intramuscular (IM) testosterone injections during infancy or childhood at a median age of 6 months (IQR: 10.3 months) to stimulate penile growth (Table 2). In six participants, the clinical effect was documented: two out of the six experienced little to no response to treatment. Exact measurements were mostly lacking.

**Table 2.** Presentation and prepubertal management.

Genitals at first presentation	
Stretched phallic structure length (mean ± SDS)	33.2 ± 9.8 mm (n = 12)
Micropenis	9/34 (26.5%)
Meatus urethrae	Top of phallus: 33/35 (94.3%)
	Glandular hypospadias: 1/35 (2.9%)
	Epispadias 1/35 (2.9%)
Right testicular remnant	Impalpable: 32/35 (91.4%)
	Inguinal: 1/35 (2.9%)
	Scrotal: 2/35 (5.7%)
Left testicular remnant	Impalpable: 32/35 (91.4%)
	Inguinal: 2/35 (5.7%)
	Scrotal: 1/35 (2.9%)
Labioscrotal fusion	Fused: 34/35 (97.1%)
	Posterior fusion: 1/35 (2.9%)

Diagnosis and follow-up during childhood	
Age at first presentation [median (IQR)]	1.2 years (2.5)
	Range: 0–14 years (n = 35)
Diagnosis	Surgery: 12/35 (34.3%)
	AMH: 8/35 (22.9%)
	hCG stimulation test: 10/35 (28.6%)
	Multiple: 5/35 (14.3%)
Additional tests to confirm diagnosis	15/30 (50.0%)
Childhood testosterone treatment	9/34 (26.5%)
Age at childhood testosterone treatment [median (IQR)]	6.0 (10.3) months (n = 9)
Dose (Sustanon^®^: 25 mg/month)	3 doses: n = 7
	6 doses: n = 2*
Reported effect	Little/None: 2/6 (33.3%)
	Good: 4/6 (66.6%)
	Missing: 3/9 (33.3%)
Age at gonadectomy [median (IQR)]	1.8 years (8.58) (n = 10)
	Range: 0.3–16.5 years

SDS: standard deviation score; micropenis: Phallic structure of <2.5 cm for neonate or 2 SD below mean for age; surgery: laparoscopy or laparotomy was used to confirm the diagnosis; AMH: undetectable AMH levels confirmed the diagnosis; hCG stimulation test: an hCG stimulation test was used using the local protocol to confirm the diagnosis; multiple: multiple methods were used for the diagnosis to be confirmed; additional tests to confirm diagnosis: additional tests were used after the diagnosis was made to confirm the findings (mainly blood sampling or laparoscopy/-tomy); childhood testosterone treatment: treatment with testosterone to increase penile size during childhood; reported effect: reported effect of testosterone treatment during childhood on penile size.

* One case was documented to have received six biweekly testosterone injections.

### Puberty and beyond

At the time of data collection, the female participant had started estrogen replacement therapy at the age of 11.3 years, while 25/34 male participants had initiated TRT at a median age of 12.4 years (IQR: 0.7) (Table 3). On request of the participant who identified as non-binary, TRT was kept at low doses, whereas in all others, incremental doses of IM testosterone esters (Sustanon^®^) were tailored every 3–6 months to clinical response and serum hormone levels (Supplementary Fig. S1). Meanwhile, two adult participants had switched to long-acting testosterone undecanoate. There were 17 participants who had reached the end of puberty (Tanner G4/G5) (Table 3).

**Table 3.** Puberty and pubertal management.

Puberty at last follow-up	
Age at last follow-up (mean ± SDS)	15.0 ± 5.7 years (n = 35)
Pubertal development at last follow-up	Prepubertal: 11/35 (31.4%)
	Midpuberty: 7/35 (20.0%)
	End-pubertal: 17/35 (48.6%)

Testosterone replacement therapy	
Testosterone replacement therapy initiated	25/34 (73.5%)*
	(1 estrogen replacement therapy)
Age at start of substitution [median (IQR)]	12.4 (0.7) years (n = 25)
	Range: 10.7–16.5 years
Start dose [median (IQR)]	(12.5) mg/2 weeks

Growth	
Adult height (mean ± SDS)	176.3 ± 6.0 cm (n = 17)
Peak height velocity (mean ± SDS)	9.7 ± 1.7 cm/year (n = 19)
Total pubertal growth (mean ± SDS)	23.8 ± 6.3 cm (n = 17)
Target height reached (Delta midparental height ≥−8 cm)	15/15 (100%)

Stretched penile length	
Prepubertal SPL (mean ± SDS)	5.0 ± 1.2 cm (n = 21)
End-pubertal SPL (mean ± SDS)	9.6 ± 2.5 cm (n = 11)
Pubertal SPL increase (mean ± SDS)	4.2 ± 1.3 cm (n = 7)

SDS: standard deviation score; prepubertal: Tanner G1/G2; midpuberty: Tanner G3; end-pubertal: Tanner G4/G5; peak height velocity: PHV of all end-pubertal cases and midpubertal cases who had received TRT for at least 2 years; SPL: stretched penile length.

* Two cases were treated with long-acting testosterone undecanoate (Nebido©).

The DSD-LIFE questionnaire was completed by 11 of the 17 end-pubertal participants (Table 4). At cross-sectional evaluation, 5 of the 11 (45.5%) reported that they had suboptimal understanding of the goals and effects of TRT at the time they initiated puberty induction and recommended that treating physicians provide a more thorough explanation of the treatment details to boys who are started on TRT at a level they can understand. Furthermore, only 6 (54.5%) and 5 (45.5%) of the 11 indicated that they were well informed in the last year regarding the risks of treatment and potential side effects, respectively (Table 4).

**Table 4.** Results of the DSD-LIFE questionnaire.

Overview	
Completed questionnaires	11/17 (64.7%)
Overall satisfaction regarding TRT	Satisfied: 10/11 (90.9%)
	Neutral: 1/11 (9.1%)

Early treatment	
Age information was first given	4–12 years: 9/11 (81.8%)
	13–17 years: 2/11 (18.2%)
I understood the purpose of TRT	Good: 6/11 (54.5%)
	Neutral: 4/11 (36.4%)
	Insufficient: 1/11 (9.1%)
I understood the information that was given	Good: 10/11 (90.9%)
	Neutral: 1/11 (9.1%)

Information received in the last 12 months	
Regarding my condition	Satisfied: 8/11 (72.7%)
	Neutral: 2/11 (18.2%)
	Dissatisfied: 1/11 (9.1%)
Regarding my treatment	Satisfied: 10/11 (90.9%)
	Neutral: 1/11 (9.1%)
Regarding my treatment options	Satisfied: 7/11 (63.6%)
	Neutral: 3/11 (27.3%)
	Dissatisfied: 1/11 (9.1%)
Regarding the risks of treatment	Satisfied: 6/11 (54.5%)
	Neutral: 3/11 (27.3%)
	Dissatisfied: 2/11 (18.2%)
Regarding side effects	Satisfied: 5/11 (45.5%)
	Neutral: 4/11 (36.4%)
	Dissatisfied: 2/11 (18.2%)

**Figure 1.**
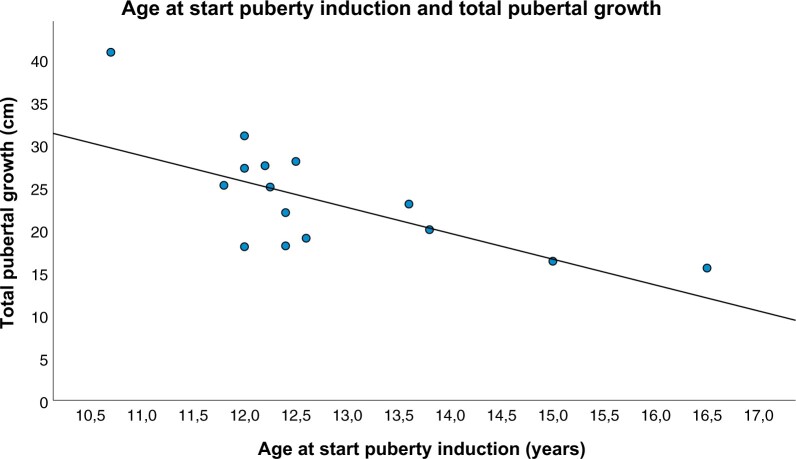
**Scatterplot of total pubertal growth and age at start of puberty induction**.

All end-pubertal participants had a final height within their target height range and had experienced adequate total pubertal growth (Table 3). In addition, adequate PHV was seen in all end-pubertal and midpubertal participants who had received TRT for at least 2 years (Table 3 and Supplementary Fig. S2). The age at the start of TRT showed a negative correlation with the total pubertal growth (Fig. 1) but no correlation with PHV (ρ = −0.642, *P* = 0.007 and ρ = −0.315, *P* = 0.203, respectively), delta target height or adult height (NS).

Implantation of testes prostheses was only discussed in two out of five centers with end-pubertal participants. Of those who had received information on this possibility, 9 out of 10 (90%) opted to undergo this intervention, at a mean age of 16.7 ± 1.0 years (range: [15.4–18.7 years]). Testes prostheses had been *in situ* for a median duration of 2.3 years (IQR: 8.0 years) and had not led to complications in any of the participants. In boys, the mean and median SPL at the end of puberty were 9.6 ± 2.5 and 9.5 cm (IQR: 1.5 cm), respectively. Five out of 11 participants (45.5%) had an SPL of ≤9 cm, corresponding to −2.5 SD at adult age. Measurements before and after puberty were available in seven participants. These participants had a mean pubertal SPL increase of 4.2 ± 1.3 cm (Table 3).

### Genetics

DNA quality of one participant was suboptimal and was therefore excluded from further analysis. The results of the DSD-WES panels of the 34 remaining participants are presented in Table 5. In three unrelated participants, heterozygous (likely) pathogenic variants were identified in *DHX37*. Participants 1 and 2 carried the same missense variant, c.923 G > A (p.Arg308Gln). In Participant 3, a different missense variant was identified, c.1000C>T (p.Arg334Trp).

**Table 5.** Summary of withheld variants.

(Likely) Pathogenic variants									
	Phenotype	Gene	Transcript	HGVS transcript	HGVS Protein	rsID	REVEL score	**GnomAD v2.1.1** **VAF**	**GnomAD v2.1.1** **Homozygotes**
Case 1	BTR, Mic	*DHX37*	NM_032656.4	c.923G>A	p.(Arg308Gln)	rs1384892917	0.451	0.00003187	0
Case 2	BTR, Mic, pSF	*DHX37^1^*	NM_032656.4	c.923G>A	p.(Arg308Gln)	rs1384892917	0.451	0.00003187	0
Case 3	BTR, Mic	*DHX37^2^*	NM_032656.4	c.1000C>T	p.(Arg334Trp)	–	0.595	–	–

Variants of unknown significance									
	Phenotype	Gene	Transcript	HGVS transcript	HGVS Protein	rsID	REVEL score	**GnomAD v2.1.1** **VAF**	**GnomAD v2.1.1** **Homozygotes**
Case 4	BTR	*SOX8*	NM_014587.5	c.200G>A	p.(Cys67Tyr)	–	0.668	–	–
Case 5	BTR	*WT1*	NM_024426.6	c.1063T>C	p.(Cys355Arg)	rs142059681	0.807	0.0005373	0
Case 6	BTR	*PROKR2*	NM_144773.3	c.868C>T	p.(Pro290Ser)	rs149992595	0.939	0.0001343	0

BTR: bilateral testicular regression; Mic: micropenis; pSF: posterior scrotal fusion; 1: segregation analysis revealed maternal inheritance; 2: segregation analysis revealed *de novo* variant. REVEL score: rare exome variant ensemble learner; VAF: variant allele frequency; homozygotes: reported number of homozygotes.

Heterozygous missense variants of unknown significance in *SRY-Box Transcription Factor 8* (*SOX8*), *Wilms' tumor 1* (*WT1*), and *Prokineticin Receptor 2* (*PROKR2*) were identified in three other participants (Participants 4–6). These rare variants had likely deleterious effects on protein function, based on *in silico* predictions (REVEL score). Case 5 underwent thorough nephrological screening which did not reveal any renal abnormalities. ORVAL analysis did not reveal any (likely) pathogenic, oligogenic variant combinations that could be linked to the phenotype.

### Pathology

Gonadal remnants of eight participants without a *DHX37* variant were analyzed. Findings were similar in all cases, revealing fibrous connective tissue, fat, and an absence of gonadal cells. In some, a blind-ending spermatic duct was visible in the resected tissue, but there was no uterus or fallopian tube.

**Figure 2.**
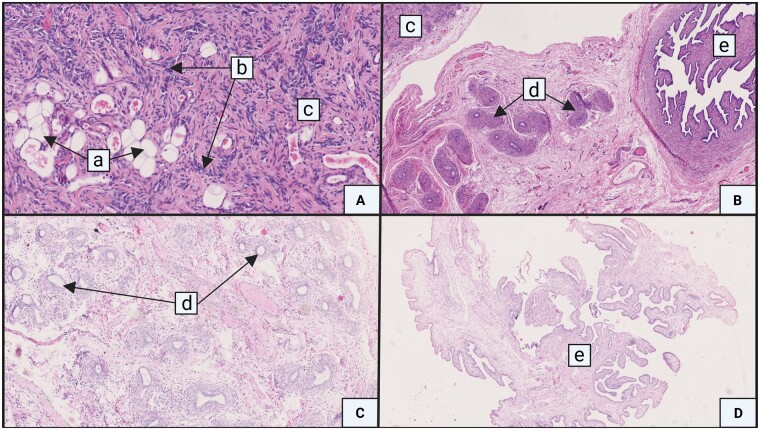
**Gonadal remnants and adnexes obtained at laparoscopic exploration.** (**A** and **B**) Participant 2 and (**C** and **D**) Participant 3. Samples were stored as formalin-fixed paraffin-embedded material and stained with hematoxylin/eosin. Magnification ×50. a: fat tissue; b: sex chords; c: gonadal tissue; d: epididymis; and e: fallopian tube.

Gonadal remnants of Participants 2 and 3, with *DHX37* variants, were available for analysis. In Participant 2, the left gonadal remnant showed, apart from a large portion of fibrotic tissue, a small area of undifferentiated gonadal tissue, containing sex cords in a background of gonadal stroma, an absence of germ cells, and fat infiltration, next to a well-developed epididymis and fallopian tube. The right side showed an epididymis and fallopian tube, but no gonadal tissue (Fig. 2A and B). In Participant 3, the left side revealed an epididymis and a fallopian tube. A mesonephric rest and a fallopian tube were visible on the right side (Fig. 2C and D). In neither of these patients were germ cells found.

### Participants with *DHX37* variants

An overview of clinical data of Participants 1–3 is presented in Table 6.

**Table 6.** Details of participants with DHX37 variant.

Presentation	Participant 1	Participant 2	Participant 3
Gestational complications	None	Twin pregnancy*	Cytomegalovirus (CMV) infection
		Placental abruption^+^	
		Preterm 34 weeks 3 days	
Age at presentation	Neonatal	1.0 year	2 days
Genitalia at presentation	Micropenis, SPL 15 mm and penile width 5 mm Orthotopic meatus Fused scrotum No palpable testes	Micropenis, not measured but judges as ‘severe’ by clinician Epispadic meatus Posterior labioscrotal fusion No palpable testes	Micropenis, not measured but judges as ‘severe’ by clinician Orthotopic meatus No palpable testes

Early management	Participant 1	Participant 2	Participant 3
Testosterone treatment	Yes	No	Yes
Age start	2 months	NA	8 months
Regimen	Sustanon^®^ 50 mg/month	NA	Sustanon^®^ 25 mg/2 weeks
	6 doses		6 doses
			DHT creme daily
	DHT creme daily		
	2 months		2 months
Effect	Little effect: SPL 30 mm and width 10 mm	NA	SPL 20 mm, no growth
Gender identity	Male	Female	Non-binary

Puberty induction	Participant 1	Participant 2	Participant 3
Age start	12.5 years	11.3 years	13.7 years
Regimen	Incremental Sustanon^®^	Daily estradiol 200 µg	Incremental Sustanon^®^
Time on HRT	1.5 year	NA	2.9 years
Tanner at last visit	G3P3A2	Prepubertal	G3P3A2
Growth since start HRT	12 cm	NA	17.9 cm
Peak height velocity	9 cm/year	NA	9.9 cm/year
SPL at start HRT	30 mm	NA	35 mm
SPL at last visit	57 mm	NA	56 mm

* Dizygotic twin.

+ Placental abruption at gestational age of 3 months.

Sustanon©: intramuscular injections of testosterone esters; NA: not applicable; HRT: hormone replacement therapy; SPL: stretched penile length.

Participant 1 was referred to the pediatric endocrinology department soon after birth because of a micropenis (SPL 1.5 cm, penile with 0.5 cm), bilateral absent testicles, and undetectable AMH levels. He received testosterone treatment between the age of 2 and 8 months to increase penile size, which had little effect. At puberty, TRT resulted in adequate statural growth; however, penile growth remained suboptimal. The *DHX37* variant was absent in the mothers’ DNA, but paternal DNA was not available for research.

Participant 2 had presented at the age of 1.0 year with a diagnosis of 46,XY DSD of unknown etiology. She was born from a twin pregnancy (dizygotic), complicated by placental abruption during the third month of the pregnancy. She had posterior labioscrotal fusion, a phallic structure with minimal epispadic meatus, and bilateral absence of gonads. She was raised as a girl and later reported to have a female gender identity. At the age of 11.3 years, puberty induction was started with incremental estradiol doses. Segregation analysis revealed maternal inheritance of the *DHX37* variant.

Participant 3 was diagnosed soon after birth with a severe micropenis and bilateral absent testes. At the age of 8 months, testosterone treatment was given without any effect on penile size. During childhood, unilateral neurosensorial deafness occurred which was attributed to a gestational cytomegalovirus infection. Puberty induction with incremental doses of testosterone resulted in good statural growth; however, penile growth remained suboptimal. Segregation analysis revealed that the identified *DHX37* variant was *de novo*.

## Discussion

Long-term outcome studies of individuals born with BTR are currently lacking, hampering counseling of patients and parents. Therefore, this study assessed the initial diagnostic approach and management during childhood and puberty of individuals born with BTR. The underlying causes of BTR were sought through careful retrospective evaluation of important gestational and perinatal events, histological analysis of gonadal rests, and exome-based panel testing of genes (n = 241) involved in gonadal development and spermatogenesis.

Severe gestational and/or obstetric complications were documented in 50% of our participants. Remarkably, five participants were born from separate, multiple pregnancies (14.7%), of which three were part of monozygotic twin pairs with documented twin-to-twin transfusion. None of the three monozygotic twin pairs were concordant for the TR phenotype. These data suggest that BTR might be associated with unfavorable fetal and perinatal conditions, and support the environmental hypothesis as one of the main causes of BTR.

**Figure 3.**
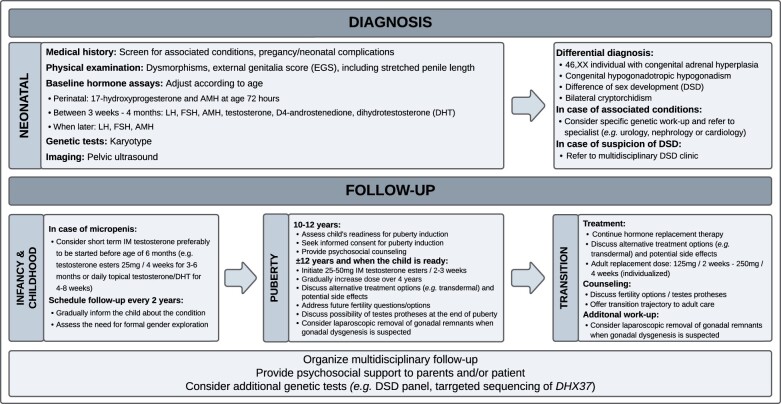
**Flowchart: management of bilateral testicular regression**.

In line with other studies, multiple tests were used to confirm the diagnosis of BTR, underscoring that the diagnostic work-up of this condition needs standardization (Heksch *et al.*, 2019) and that it has evolved over time, with less surgery and more reliance on AMH dosage over the years. Based on our experience, we propose a thorough physical examination, including a precise genital description using the EGS (Van Der Straaten *et al.*, 2020), baseline hormone assessment (including gonadotropins and AMH levels), karyotyping and ultrasound, and only in selected cases, hCG stimulation tests and/or surgical exploration, depending on the patient’s phenotype and age (Fig. 3).

Of note, two participants with marked undervirilization at birth (and female sex assignment in one) did not have a typical male gender identity. Therefore, a multidisciplinary approach, including a child psychologist with experience in gender development, is required during follow-up in childhood, and most importantly, prior to the start of HRT.

During physiological puberty, boys grow on average 25–30 cm with a PHV of 9.5 cm/year (Tanner and Davies, 1985; Wood *et al.*, 2019). In a study by Fouatih *et al.* (2019), a final height within their target height range but below average pubertal growth was reported in nine boys with BTR who received TRT. In line with these findings, all 15 end-pubertal participants in our study had an adult height within their adult target height range. A negative correlation between pubertal growth and the age at start of TRT was found, as seen in individuals with constitutional delay of puberty (Tanner and Davies, 1985). A recent guideline suggests to induce puberty using an initial IM testosterone dose of 25–50 mg monthly starting from as early as 10 years and gradually increasing the dose until an adult dose of 150–250 mg every 2–4 weeks is reached (Nordenström *et al.*, 2022). In our study, all participants had received similar incremental doses. Importantly, 45.5% of end-pubertal participants reported that they had suboptimal comprehension of the goals and outcomes of this treatment when the treatment was started, highlighting the importance of providing sufficient and comprehensive information to hypogonadal boys who need puberty induction (Fig. 3).

The mean SPL at the end of puberty was 9.6 ± 2.5 cm. In 5 out of 11 adult participants (45.5%), the SPL was 2.5 SD or more below the average for adult (Belgian) males (i.e. <9 cm), which corresponds to the definition of micropenis in adulthood (Tsang, 2010; Tack *et al.*, 2022). A mean increase of 4.2 ± 1.3 cm was documented during puberty and this reflects suboptimal penile growth (Tomova *et al.*, 2010). Of note, only four of the nine participants with a documented micropenis at presentation had reached Tanner G4/G5 at the time of the study. During childhood, the penis grows significantly, especially in the first year of life during mini-puberty (Camurdan *et al.*, 2007; Pasterski *et al.*, 2015). Boys with BTR who do not receive TRT in this period lack this postnatal exposure to androgens, which could play an important role in the smaller adult SPL. Hormonal treatment during mini-puberty is debated because of the lack of involvement of the child in his therapeutic plan at this young age (Johnson *et al.*, 2023). However, boys without a (likely) pathogenic *DHX37* variant showed a good penile growth response to IM testosterone administration during infancy/childhood. It is possible that this treatment has resulted in a longer adult SPL in these boys (Zenaty *et al.*, 2006). However, as exact measurements were often lacking and only nine participants received childhood testosterone treatment, these results need to be interpreted with caution. Boys with a *DHX37* variant experienced minimal penile growth during childhood testosterone treatment and exhibited suboptimal penile growth after starting TRT. Long-term studies are warranted to study the effects of testosterone treatment during mini-puberty and infancy on penile size.

In our study, most young adult men (90%) opted for implantation of testis prostheses when this option was discussed with them, which was not always the case. These differences between centers emphasize the need for the development of clinical practice guidelines to offer standardized care to individuals with BTR, as testes prostheses could improve the patient’s psychosexual well-being (Heksch *et al.*, 2019).

Two heterozygous (likely) pathogenic *DHX37* variants were identified in three participants (8.8%). The incidence of *DHX37* missense variants was lower in our study as compared to 4/16 (25%) with BTR reported by McElreavey *et al.* (2020). This can partly be explained by the *a priori* exclusion of boys with severe forms of hypospadias in our cohort (Zidoune *et al.*, 2021). On the other hand, in our recent study reporting on the genetic etiology of hypospadias in 99 cases, no (likely) pathogenic *DHX37* variants were identified (Tack *et al.*, 2022). *DHX37* codes a DNA helicase, and both missense variants alter a highly conserved arginine that plays an important role in the DNA/RNA contact interface and leads to severe phenotypes (McElreavey *et al.*, 2020). The first missense variant, p.Arg308Gln, was found in Participants 1 and 2, who are unrelated. This variant has been reported in cases with phenotypes ranging from typical females due to complete gonadal dysgenesis, to males with BTR and micropenis (Buonocore *et al.*, 2019; da Silva *et al.*, 2019; McElreavey *et al.*, 2020). Participant 3 had a missense variant, p.Arg334Trp, which has been reported previously by McElreavey *et al.* (2020) in a case with a similar phenotype, except for the presence of hypospadias in the latter. For Participant 1, no gonadal histology was available; in Participant 2, the gonads had almost completely been replaced by fibrous tissue, apart from a unilateral small area of undifferentiated gonadal tissue. Histological analysis in Participant 3 revealed the absence of gonadal tissue. Of note, both Participants 2 and 3 had fallopian tubes on histological examination. It is plausible to hypothesize that in these cases, BTR occurred secondary to gonadal dysgenesis, as has been reported in other forms of DSD, e.g. 45,X/46,XY DSD (Cools *et al.*, 2011). The shorter SPL in Participants 1–3, with DHX37 missense variants and the presence of Müllerian remnants in Participants 2 and 3, suggests earlier gonadal regression than in the other participants. In the eight other participants with available gonadal histology, the TR had occurred after the regression of Müllerian structures under the influence of testicular AMH in early pregnancy, highlighting the different etiology and developmental pathway of these cases. In our cases with BTR, no germ cells were found in the small undifferentiated gonadal tissue area. In a study by He *et al.* (2022), germ cells were found in 3.1% (16/513) of cases with unilateral TR and were all negative for Oct3/4 staining. However, data regarding genital virilization, genetic tests, and presence of Müllerian remnants were not reported in this study. Based on these findings, we would propose planning resection of gonadal remnants only in cases where gonadal dysgenesis is suspected, due to the risk for gonadal germ cell cancer. In patients with the typical presentation of BTR, i.e. bilateral absence of functional testes in an otherwise typically virilized male neonate, resection can be considered during other surgeries, e.g. implantation of testes prostheses.

Remarkably, both male participants carrying a *DHX37* variant showed little growth response to TRT during infancy or puberty to increase penile size. At Tanner G3, SPL remained suboptimal (i.e. 56 and 57 mm) in both participants, in contrast to their adequate pubertal growth spurt, showing a good response to TRT. This supports the idea of an inverse correlation of penile growth potential with the timing of penile developmental arrest during intra-uterine life.

The strengths of this study included the use of genetic, histological, and longitudinal clinical data in one of the largest cohorts of individuals with BTR reported to date. Weaknesses included the missing data in several participants and the use of different methods to measure hormone levels in different laboratories at diagnosis which did not allow pooled analysis. Furthermore, the small sample size due to the rarity of the condition warrants caution.

In conclusion, BTR is a heterogenous condition, with different etiologies, and in which the gonads can regress early or later during fetal development. Diagnosis and management of BTR can be difficult as clinical guidelines are currently lacking. Incremental doses of testosterone to induce and support puberty provide adequate pubertal statural growth and virilization, albeit with suboptimal adult penile length in half of the patients. Testosterone treatment should not be started before the boy has a clear understanding of the goals and effects of puberty induction so that he can be involved in the decision to start TRT. Early TRT, mimicking physiologic androgen exposure during infancy, could be beneficial for postnatal penile development, although this may depend on the timing of TR, and hence, arrest of penile development.

## Supplementary Material

hoad047_Supplementary_Data

## Data Availability

The data underlying this article will be shared on reasonable request to the corresponding author.
