# Supplementary material for: Etiology, histology, and long-term outcome of bilateral testicular regression: a large Belgian series
Source: Hum Reprod Open. 2023 Dec 1;2023(4):hoad047. doi: 10.1093/hropen/hoad047 (PMC11631441; doi:10.1093/hropen/hoad047)
Supplement: hoad047_Supplementary_Data [file hoad047_supplementary_data.zip › HRO-23-0144-R2-SuppTables1-2.docx]

**Supplementary Table S1.** Overview of used statical methods

| **Whole exome sequencing** | The SureSelectXT Low Input Human All Exon V7 kit (Agilent Technologies) was used for exome enrichment, followed by paired-end sequencing on a NovaSeq 6000 (2x150 bp cycles; Illumina). Read alignment and variant calling were performed using an in-house developed bcbio-based pipeline. |
| --- | --- |
| **Filtering of variants** | Variants were interpreted and filtered using in-house software (Seqplorer).  Criteria:  Exclusion of:   - - 3’ UTR & 5’ UTR variants   - Downstream & Upstream gene variants   - Intronic variants   Non-coding transcript variants  Synonymous variants  Variant allele frequency:  gnomAD v2.1.1: <0.05  Variants in known recessive genes:  Repeated analysis without restriction of allele frequency to exclude compound heterozygosity  Final interpretation, combination of following factors:  Population frequency  In silico predictions:  REVEL score: Missense variants  ADA and RF score: Splice site variants  VarSome & ClinVar variant classification  Expected phenotype based on literature search |
| **Segregation analysis** | Sanger sequencing* |
| **Oligogenic resource for variant analysis (ORVAL)** | Filtered exome data were inputted in the Oligogenic Resource for Variant AnaLysis (ORVAL) online platform ^1^.  Filtering was performed as follows:  1.   Filter on mendeliome gene positions  2.   Remove homozygous wild type entries and uncalled genotype entries  3.   Annotate with gnomADv2.1 and filter on AF <0.02  4.   Remove low and medium impact mendeliome variants of genes not included in supplemental table 1C (cfr. infra). Following variants were removed:  a. Synonymous variants  b. Missense variants  c. Splice region variants  d. 3’ UTR variants  e. 5’ UTR variants  f. Downstream & Upstream gene variants  g. Intronic variants  h. Non-coding transcript variants |

**Supplementary Table S2.** Genes included in the whole exome sequencing panel

| **Whole exome sequencing-based gene panel** ^2,3^ | | | | | |
| --- | --- | --- | --- | --- | --- |
| *AARS2* | *CHD7* | *FGF9* | *LHCGR* | *PKD1* | *SPO11* |
| *ABCA1* | *CLPP* | *FGFR1* | *LHX1* | *PLCZ1* | *SPRY4* |
| *ADGRG2* | *CREBBP* | *FGFR2* | *LHX3* | *PLK4* | *SRA1* |
| *AK7* | *CYB5A* | *FIGLA* | *LHX4* | *PLXNA1* | *SRD5A2* |
| *AKAP4* | *CYP11A1* | *FLNA* | *LRRC6* | *PMFBP1* | *SRY* |
| *AKR1C2* | *CYP11B1* | *FLRT3* | *MAGEB4* | *PNPLA6* | *STAG3* |
| *AKR1C4* | *CYP17A1* | *FOXL2* | *MAMLD1* | *POF1B* | *STAR* |
| *AMH* | *CYP19A1* | *FSHB* | *MAP2K2* | *POLR2C* | *STK36* |
| *AMHR2* | *CYP21A2* | *FSHR* | *MAP3K1* | *POLR3A* | *STX2* |
| *ANOS1* | *DCC* | *FSIP2* | *MC4R* | *POR* | *SUN5* |
| *APOA1* | *DGKK* | *GALNTL5* | *MCM8* | *PROK2* | *SYCE1* |
| *AR* | *DHCR7* | *GAS8* | *MCM9* | *PROKR2* | *SYCP3* |
| *ARX* | *DHH* | *GATA4* | *MEI1* | *PROP1* | *TAC3* |
| *ATF3* | *DHX37* | *GDF9* | *MEIOB* | *PSMC3IP* | *TACR3* |
| *ATRX* | *DMC1* | *GH1* | *MID1* | *RBMXL2* | *TAF4B* |
| *AURKC* | *DMRT1* | *GNRH1* | *MNS1* | *REC8* | *TDRD6* |
| *AXL* | *DMXL2* | *GNRHR* | *MRPS22* | *RELN* | *TDRD7* |
| *BMP15* | *DNAAF2* | *HAUS7* | *MSH4* | *RNF216* | *TDRD9* |
| *BMP4* | *DNAAF4* | *HDAC8* | *MSH5* | *RNF220* | *TEX11* |
| *BMP7* | *DNAAF5* | *HESX1* | *MTOR* | *RSPH1* | *TEX14* |
| *BNC1* | *DNAH1* | *HFM1* | *NANOS2* | *RSPH3* | *TEX15* |
| *BNC2* | *DNAH9* | *HHAT* | *NANOS3* | *RSPH9* | *TP63* |
| *BRAF* | *DNAI1* | *HOXA13* | *NBN* | *RSPO1* | *TRIM37* |
| *BRDT* | *DNAI2* | *HS6ST1* | *NLRP3* | *RXFP2* | *TSPYL1* |
| *BSCL2* | *DNAJB13* | *HSD17B3* | *NNT* | *SECISBP2* | *TTLL5* |
| *C11orf70* | *DNMT1* | *HSD17B4* | *NOBOX* | *SEMA3A* | *TWNK* |
| *CATSPER1* | *DNMT3B* | *HSD3B2* | *NOS1* | *SEMA7A* | *UBE2B* |
| *CATSPERE* | *DPY19L2* | *HSF2* | *NOTCH1* | *SLC26A3* | *UBR2* |
| *CBX2* | *DUSP6* | *HYDIN* | *NR0B1* | *SOHLH1* | *USP26* |
| *CCDC103* | *E2F1* | *IL17RD* | *NR2F2* | *SOHLH2* | *VAMP7* |
| *CCDC141* | *EIF4ENIF1* | *INSL3* | *NR5A1* | *SOS1* | *WDR11* |
| *CCDC155* | *EP300* | *KDM3A* | *NRAS* | *SOX10* | *WDR66* |
| *CCDC39* | *ERBB4* | *KHDRBS1* | *NSMF* | *SOX2* | *WNT4* |
| *CCDC40* | *ESR1* | *KISS1* | *NUP107* | *SOX3* | *WT1* |
| *CDC14A* | *ESR2* | *KISS1R* | *OTUD4* | *SOX8* | *WWOX* |
| *CEP135* | *FANCA* | *KLHL10* | *PANK2* | *SOX9* | *XRCC2* |
| *CEP290* | *FANCM* | *LARS2* | *PATL2* | *SPAG17* | *ZFPM2* |
| *CFAP43* | *FEZF1* | *LEP* | *PCSK1* | *SPATA16* | *ZMYND15* |
| *CFAP44* | *FGF17* | *LEPR* | *PDHA2* | *SPIDR* | *ZNRF3* |
| *CFAP69* | *FGF8* | *LHB* | *PIH1D3* | *SPINK2* | *ZPBP* |
| *CFTR* |  |  |  |  |  |

AF: allele frequency. *Sequence of primers available upon request.

**References**

1. Renaux A, Papadimitriou S, Versbraegen N, Nachtegael C, Boutry S, Nowé A, et al. ORVAL: a novel platform for the prediction and exploration of disease-causing oligogenic variant combinations. Nucleic Acids Res. 2019 Jul 1;47(W1):W93–8. Available from: 10.1093/nar/gkz437

2. Oud MS, Volozonoka L, Smits RM, Vissers LELM, Ramos L, Veltman JA. A systematic review and standardized clinical validity assessment of male infertility genes. Hum Reprod. 2019 May 1;34(5):932–41. Available from: 10.1093/humrep/dez022

3. Constitutioneel genetische aandoeningen - Centrum Medische Genetica.
