# Supplementary figures and images for: Etiology, histology, and long-term outcome of bilateral testicular regression: a large Belgian series
Source: Hum Reprod Open. 2023 Dec 1;2023(4):hoad047. doi: 10.1093/hropen/hoad047 (PMC11631441; doi:10.1093/hropen/hoad047)

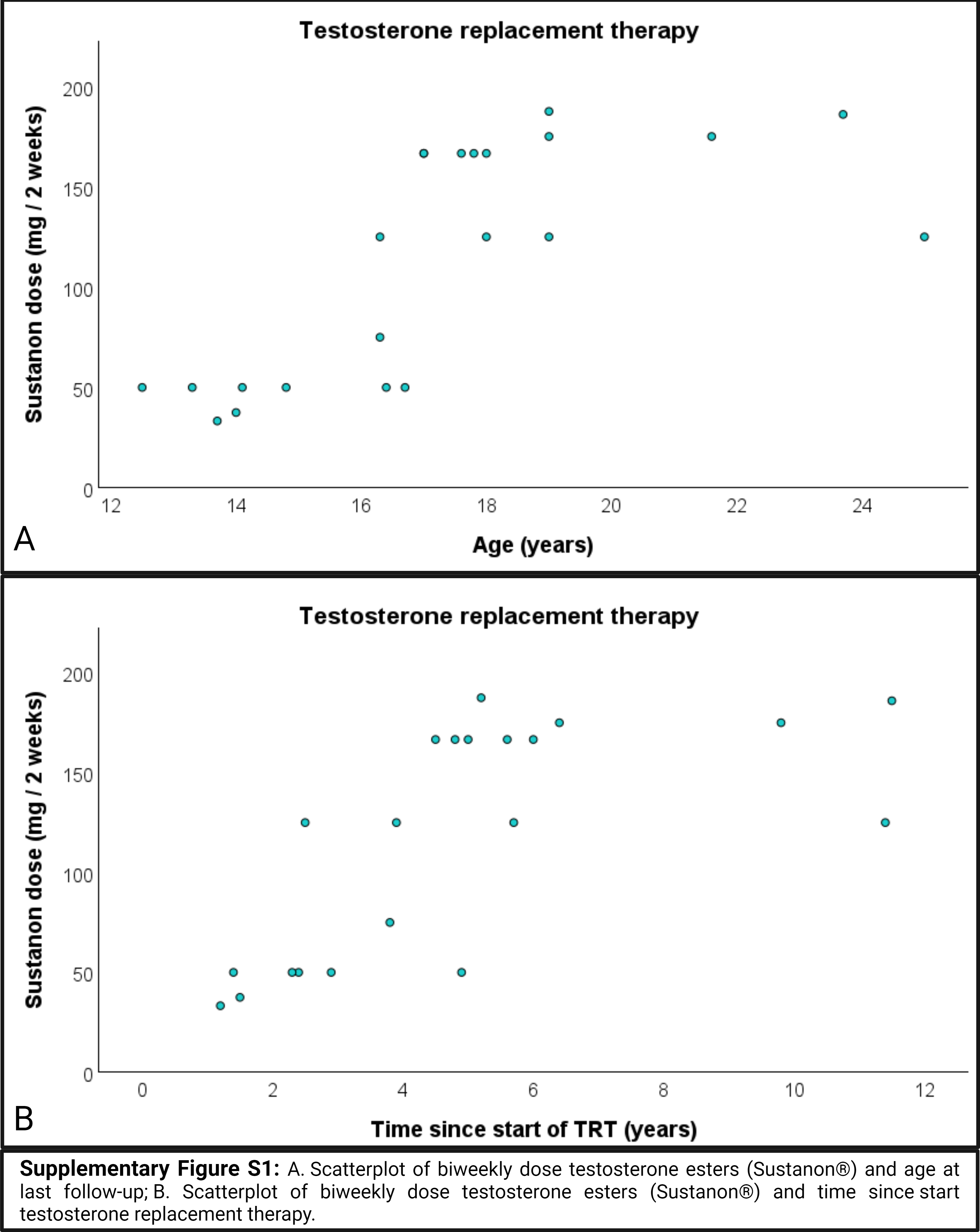

Supplement: hoad047_Supplementary_Data [file hoad047_supplementary_data.zip › Supplementary Figure S1.png]

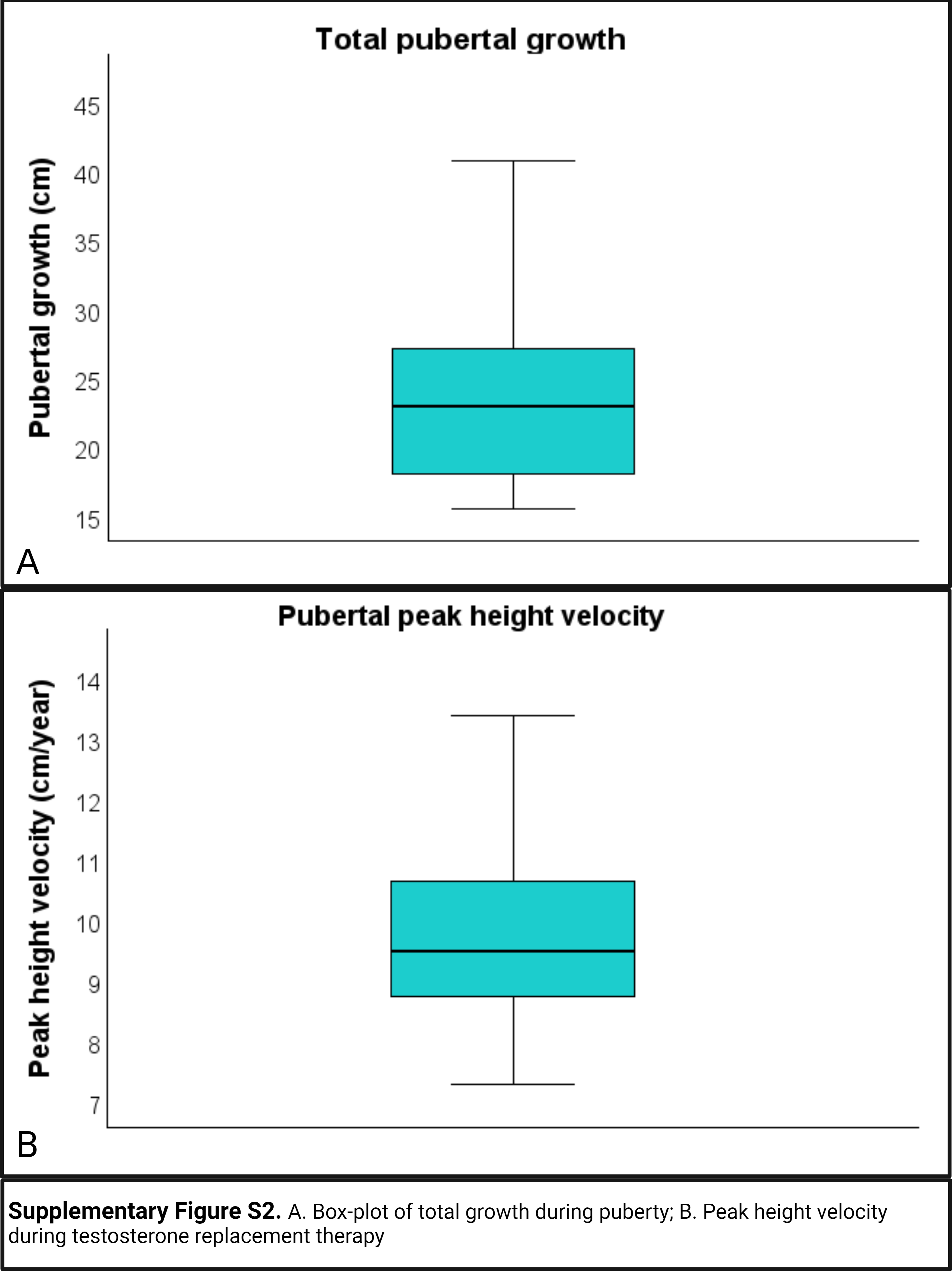

Supplement: hoad047_Supplementary_Data [file hoad047_supplementary_data.zip › Supplementary Figure S2.png]
